# Supplementary material for: paPAML: An Improved Computational Tool to Explore Selection Pressure on Protein-Coding Sequences
Source: Genes (Basel). 2022 Jun 18;13(6):1090. doi: 10.3390/genes13061090 (PMC9222883; doi:10.3390/genes13061090)
Supplement: Supplementary file 1 [file genes-13-01090-s001.zip › Descriptions of Supplementary Material.pdf]

Supplementary Data S1

Original data and results for APOL1, CEBPE, fn3_Bifidobacterium, GINS3, and LYSIN. ".fasta" provide the processed fasta sequences after gap removals. ".tre" provide the original species tree (subsequently, paPAML generates all necessary trees with individual foreground branch labels). ".results" provides the paPAML results compiled from all applied tests and subprocesses. ".fa" provides a codon-by-codon and test-by-test representation with highlighted significantly selected codons (capital letters, positive and negative selection). The ".fa" can be aligned to the original gene for site-by-site comparisons of the original genes (e.g., Supplementary Data S3). Text format.

Supplementary Data S2

Positive and negative selected sites of APOL1-4 (APOL1-4_paPAML.pde). Description: AA alignment for exon 6 and 7 (corresponding to APOL1 vA) in PDE format. Data Editor (PhyDE, http://www.phyde.de/). The paPAML results are shown for T1_Bayes_1_2, T1_Bayes_7_8, T2_Bayes, HyPhy_neg (negative selection), and HyPhy_pos (positive selection) (lines 7-11) compared to previous PAML results) (line 5). For further explanations, see Müller et al. [18]. Pde (PhyDE) format.

Supplementary Data S3

Positively selected sites for the *fn3* gene of *Bifidobacterium* with the reference species *B. adolescentis* (line 1) and derived amino acids (AA, line 2). The original gene sequence was extracted from Data_Sheet_2_Positive Selection in Bifidobacterium Genes Drives Species-Specific Host–Bacteria Communication.FASTA [25]. The analysed area of the gene (with removed variable regions via Gblocks) was extracted from Data_Sheet_7_Positive Selection in Bifidobacterium Genes Drives Species-Specific Host–Bacteria Communication.FASTA [25]. In Dyachkova et al. [25] the Branch-site model of PAML was applied. Correspondingly, we used the paPAML Branch-site model (T2_Bayes) with the settings: paPAML.pl -p 30 -d -t 2. The run finished in 35 hours. Data Editor (PhyDE, http://www.phyde.de/). Pde (PhyDE) format.
