# Supplementary material for: paPAML: An Improved Computational Tool to Explore Selection Pressure on Protein-Coding Sequences
Source: Genes (Basel). 2022 Jun 18;13(6):1090. doi: 10.3390/genes13061090 (PMC9222883; doi:10.3390/genes13061090)
Supplement: Supplementary file 1 [file genes-13-01090-s001.zip › Suplementary Table S1.pdf]

Supplementary Table S1. Results of the selection analysis. Codons numbers without any label were found by the site model. Codon numbers labelled with ($^{\Delta}$) were detected by the branch-site model and those with ($^{\Diamond}$) via the HyPhy FEL algorithm. The number of branches under positive selection detected by the branch-site model does not include branches with significant LRT results but without sites under positive selection.

Data set	Codons under significant positive selection	Codons under significant negative selection	Number of branches under positive selection
**GINS3 exonization**	2, 8, 24, 25, 26, 28, 29, 37	-	2 (branch - site model)
**CEBPE**	-	7, 13, 18, 30, 43, 51, 53, 54, 55, 56, 59, 61, 69, 72, 77, 81, 82, 88, 90, 91, 95, 101, 113, 119, 121, 124, 125, 128, 134, 136, 138, 157, 162, 166, 173, 181, 183, 186, 188, 189, 194, 200, 203, 204, 207, 210, 211, 214, 216, 217, 222, 225, 229, 232, 239, 241, 243, 249, 250, 253, 260, 267, 270, 271, 275, 276, 277, 279	22 (branch model)
**Lysin**	2, 3$^{\Delta}$, 4$^{\Delta}$, 5$^{\Delta}$, 12$^{\Delta}$, 13$^{\Delta}$, 15$^{\Delta}$, 17$^{\Delta}$, 18$^{\Delta}$, 20, 21, 24, 26$^{\Delta}$, 29, 32, 33$^{\Diamond}$, 34$^{\Delta}$, 41$^{\Delta}$, 45$^{\Delta}$, 52, 55, 56$^{\Delta}$, 58, 62, 63$^{\Diamond}$, 70$^{\Delta}$, 71, 74, 75, 85$^{\Delta}$, 86$^{\Delta}$, 89$^{\Delta}$, 91$^{\Delta}$, 93$^{\Delta}$, 94$^{\Diamond}$,95$^{\Delta}$, 96$^{\Delta}$, 97$^{\Delta}$, 98$^{\Delta}$, 101, 108, 111$^{\Diamond}$, 113$^{\Delta}$, 114, 115$^{\Diamond\Delta}$, 117$^{\Delta}$, 119$^{\Diamond}$, 120	16, 19, 27, 31, 40, 42, 43, 45, 65, 66, 72, 80, 90, 100, 105	11 (branch model), 9 (branch-site model)
**APOL1**	3, 8, 15, 36$^{\Diamond\Delta}$, 56, 88, 116, 126, 137, 146, 163$^{\Delta}$, 165$^{\Diamond}$, 168, 169, 184, 193, 203, 204, 206, 208, 209$^{\Diamond}$, 212, 228, 236, 245, 247$^{\Diamond}$, 250, 261$^{\Diamond}$, 263, 271, 294	16, 27, 107, 108, 121, 136, 138, 148, 153, 221, 279, 285	5 (branch model) 2 (branch-site model)
