# Supplementary material for: paPAML: An Improved Computational Tool to Explore Selection Pressure on Protein-Coding Sequences
Source: Genes (Basel). 2022 Jun 18;13(6):1090. doi: 10.3390/genes13061090 (PMC9222883; doi:10.3390/genes13061090)
Supplement: Supplementary file 1 [file genes-13-01090-s001.zip › Supplementary Data S1.zip!/Supplementary Data S1/CEBPE/Steffen_ms_0906.docx]

Article

**paPAML: An Improved Computational Tool to Explore Selection Pressure on Protein-Coding Sequences**

Raphael Steffen^1*^, Lynn Ogoniak^1^, Norbert Grundmann^2^, Anna Pawluchin^1^, Oliver Soehnlein^1,3^ and Jürgen Schmitz^1*^

**Citation:** Lastname, F.; Lastname, F.; Lastname, F. Title. *Genes* **2022**, *13*, x. https://doi.org/10.3390/xxxxx

Academic Editor: Firstname Lastname

Received: date

Accepted: date

Published: date

**Publisher’s Note:** MDPI stays neutral with regard to jurisdictional claims in published maps and institutional affiliations.

**Copyright:** © 2022 by the authors. Submitted for possible open access publication under the terms and conditions of the Creative Commons Attribution (CC BY) license (https://creativecommons.org/licenses/by/4.0/).

^1^ Institute of Experimental Pathology, ZMBE, University of Münster, 48149, Münster, Germany

^2^ Institute of Bioinformatics, Faculty of Medicine, University of Münster, 48149, Münster, Germany

^3^ Department of Physiology and Pharmacology (FyFa), Karolinska Institutet, Stockholm, Sweden

* Correspondence: [r_stef04@uni-muenster.de](mailto:r_stef04@uni-muenster.de) (RS), [jueschm@uni-muenster.de](mailto:jueschm@uni-muenster.de) (JS)

**Abstract:** Evolution is change over time. While neutral changes promoted by drift effects are most reliable for phylogenetic reconstructions, selection-relevant changes are of only limited use to reconstruct phylogenies. On the other hand, comparative analyses of neutral and selected changes of protein-coding DNA sequences (CDS) retrospectively tell us about episodic constrained, relaxed, and adaptive incidences. The ratio of sites with *nonsynonymous* (amino acid altering) versus *synonymous* (not altering) mutations directly measures selection pressure and can be analysed using the Phylogenetic Analysis by Maximum Likelihood (PAML) software package. We developed a CDS extractor for compiling protein-coding sequences (CDS-extractor) and parallel PAML (paPAML) to simplify, amplify, and accelerate selection analyses via parallel processing, including detection of negatively selected sites. paPAML compiles results of site, branch, and branch-site models and detects site-specific negative selection with the output of a codon list labelling significance values. The tool simplifies selection analyses for casual and inexperienced users and accelerates computing speeds up to the number of allocated computer threads. We then applied paPAML to examine the evolutionary impact on a new GINS Complex Subunit 3 exon, and neutrophil-associated- as well as lysin- and apolipoprotein genes. Compared with codeml (PAML version 4.9j) and HyPhy (HyPhy FEL version 2.5.26), all paPAML test runs performed with 10 computing threads led to identical selection pressure results, while total selection analysis via paPAML, including all model comparisons, was about 3 to 5 times faster than the longest running codeml model and about 7 to 10 times faster than the entire processing time of these codeml runs.

**Keywords:** codeml; paPAML; CDS-extractor; positive selection; purifying selection; PAML; HyPhy; retrotransposon exonization; neutrophil associated gene; abalone sperm lysin; apolipoprotein

**1. Introduction**

Germline mutations leave behind heritable changes, and natural selection acts on such variations within populations. The protein-coding DNA sequences (CDS) of genes, representing ~1.5% of the human genome [1], are especially exposed to natural selection. CDS are highly variable, mainly due to alternative splicing of exons. Interspersed by introns, the exons of a gene are concatenated during splicing of the precursor messenger RNA and are usually flanked by their untranslated sequence regions (UTR). CDS are structured in triplets, called codons, which encode the translation to amino acids. (Figure 1).

**Figure 1.** Gene structure, conservation, and DNA to amino acid translation. Exons (including terminal UTR) and introns are assembled to form genes (regulatory regions are not shown). After splicing of introns, mRNA triplets forming the CDS are translated into amino acids according to the amino acid code (right; codon wheel from 123RF.com, 24366216). The impact of a point mutation in a codon depends on its position. 96%, 100%, and 33% of changes at the first, second, and third codon positions, respectively, result in amino acid changes (nonsynonymous changes dN) as opposed to synonymous changes (dS), which do not affect the amino acid sequences/protein translation. A dN/dS ratio (omega ⍵) of 1 indicates the neutral evolution of the protein. Values lower than 1 suggest negative purifying selection and above 1 positive or adaptive selection.

It is necessary to analyse changes in all codon positions to determine the evolutionary impact of mutations. 96%, 100%, and 33% of possible substitutions at the first, second, or third triplet positions, respectively, lead to an exchange of amino acids (nonsynonymous changes, dN) [2,3]. Because several codons (see codon wheel in Figure 1) represent the same amino acid, described as redundancy of the genetic code, the remaining silent or synonymous changes (dS, ~4% of the first and ~67% of the third codon position) leave proteins unchanged. They are fixed in a population via drift effects in the absence of selective forces.

Nonsynonymous codon substitutions do affect the corresponding amino acid sequence. Depending on functional constraints, they are usually under selection pressure, whether positive (adaptive selection) or negative (purifying selection). This suggests that the dN to dS substitution ratio, or omega (ω), is an indication of the respective selection pressures on sequences: If dN substitutions are prevalent (ω > 1), positive selection can be inferred. In contrast, ω < 1 indicates purifying selection. ω = 1 means that substitutions of both types are more or less equally distributed [4,5], an indicator of neutral evolution. It should be mentioned that positive selection is almost always restricted to functionally critical codons at specific evolutionary periods and is possible throughout all branches of a phylogenetic tree.

A commonly used tool to determine dN/dS ratios and therefore selection pressure on multiple orthologous protein-coding sequence regions (evolving from the same ancestors) during phylogenetic comparisons is the Phylogenetic Analysis by Maximum Likelihood (PAML) suite (version 4.9j [6]). The integrated codeml analysis evaluates various hypotheses by comparing nested statistical models via likelihood ratio tests (LRTs). Of specific interest for selection analysis are (1) the site models for detecting sites under positive selection, (2) branch-site models for detecting branches and sites under positive selection, and (3) branch models for detecting branches under positive selection as embedded in PAML. For the phylogenetic scope and additional details of the tests, see Figure 2. None of the three can detect negative selection.

**Site models:** The site models allow the ratio of ω to vary among sites (e.g., codons in DNA sequences or amino acids in proteins). The models include pairs of individual, informative comparisons for applying subsequent LRT of positive selection. One model pair consists of the so-called M1a and M2a models. M1a assumes nearly neutral selection and fixes the upper bounds of ω values at 1 (ω ≤ 1), while M2a allows for positive selection (ω ≤ 1 and ω > 1) [4,7] (see also PAML User Guide Version 4.9j, Table 2. Parameters in the site models). The second model pair consists of M7 and M8. M7 assumes that ω is beta-distributed among sites (interval 0,1) and correspondingly does not allow for positively selected sites, while M8 adds an extra class of sites that allows positive selection (ω > 1) [5,7]. The M1a/M2a comparison is described as being more robust and less powerful than the M7/M8 comparison (see PAML User Guide Version 4.9j, page 29). A site is inferred to be positively selected if M2a or M8 are significantly more likely than M1a or M7. Codeml also calculates posterior probabilities of site classes based on the Bayes empirical Bayes (BEB) test [7], which can identify specific sites under positive selection if the LRT of the M1a/M2a or M7/M8 comparison is significant.

**Branch-site models:** The branch-site models can identify positively selected sites along particular branches of a given phylogeny by allowing the ω ratios to vary among these branches and sites. Like the branch models, the LRT compares the branch site [7,8] with a corresponding null model with fixed omega (ω = 1). As with the site models, a BEB test calculates posterior probabilities of specific sites under positive selection. Similar to the branch models, the branch-site models require a set of identical trees with all foreground branches labelled.

**Branch models:** The branch models allow for variation of ω values among phylogenetic branches, enabling the inference of positive selection acting on specific ancestral or terminal lineages using several ω ratios (model = 2 [9,10]. A null model that fixes the omega value at 1 (ω = 1), which implies neutral selection across the phylogeny, is compared via LRT with a model allowing omega to vary (ω > 1). Any branch or node in which the null model is significantly less likely than its counterpart is inferred to be under positive selection pressure.

PAML and its integrated codeml analysis has become one of the most used tools for testing evolutionary selection hypotheses. All three tests are combined to find evidence for adaptive selection across a sequence and its phylogeny. However, along with other limitations, codeml is restricted to running on a single processor core and is therefore very time consuming, especially when comparing considerable numbers of species or longer sequences. The tool also does not automatically calculate p-values for the model likelihood comparisons. Its output can be confusing, especially for inexperienced users, as it does not highlight the essential results or present them comprehensively. Moreover, due to the models' restrictions, PAML cannot detect site-specific negative selection, which is a frequent problem in selection analysis programs that are mostly focused on positive selection analyses and adaptive evolution. Furthermore, it requires the preparation of separate configuration files for each alignment and test, making high-throughput analyses of multiple sequences even more arduous. There are some alternatives to codeml, such as HyPhy [11].

**HyPhy FEL:** The HYpothesis testing suite using PHYlogenies (HyPhy [11]) embeds the Fixed Effects Likelihood (FEL) algorithm, which also uses ML to infer dN/dS substitution rates on a per-site basis, and it is not restricted to positive selection [12]. However, HyPhy also assumes that selection pressure is constant along the entire phylogeny and still needs to be run for one alignment and test at a time.

**Figure 2.** Phylogenetic scope of different tests. The site models and HyPhy FEL calculate significant (*) positively and negatively selected sites, respectively, averaged over all branches (labelled blue). The branch-site models combine site-specific and branch-specific positive selection analyses for all possible foreground branches (two are labelled orange). The branch models use all possible foreground branches (two are labelled green) to calculate the average ω over all codons (labelled 1 2 3 codon positions). The default settings for the codeml ctl files are given below the corresponding models.

Both codeml and HyPhy require alignment files containing distinct orthologous sequences (for example, CDS excluding the stop codon) of multiple species or populations presenting the outgroup first and their complete phylogeny as a tree file (Newick format). To easily accomplish this, we first developed the CDS-extractor using Biopython to automatically retrieve NCBI-deposited orthologous sequences and genes for various species. To make selection analyses of protein-coding alignments fast and easy, especially for large datasets, and their results more comprehensive, we further designed parallel PAML (paPAML), a Perl-based application to run codeml and HyPhy FEL calculations in parallel. We selected the Perl programming language to provide a capable and long-lasting tool that does not interfere with new versions of that language, compared to, for example, the well-known incompatibility between versions of python.

To demonstrate the evolutionary impact of novel exon cassettes (e.g., those derived from exonized *Alu* elements), we then applied the CDS-extractor and paPAML tools to comparisons of specific mobile retrotransposed *Alu* elements. *Alu*s are Short Interspersed Nuclear Elements (SINEs) that occupy more than 10% of the human genome [1]. In addition to their well-known property as neutral phylogenetic markers in primates (e.g., Hartig et al. [13]), they constitute a vital initiator of evolving CDS by integrating into genes and using the host's splicing system to add novel CDS (e.g., Schrader and Schmitz [14,15]). Neutrophil-associated genes are well-known for their adaptive capacity [16] and served as another example to test paPAML. We also analyzed the classical abalone sperm lysin sequence dataset that contains verifiable amino acid sites under positive and purifying selection [17]. The last example refers to an apolipoprotein family (APOL) that recently was investigated for adaptive evolution [18].

**2. Materials and Methods**

*2.1. CDS-extractor and Alignment*

The SeqIO and Entrez packages of Biopython (python 3.7 [19]) were used to design the CDS-extractor. The program automatically retrieves gene orthologs derived from the NCBI Eukaryotic Genome Annotation Pipeline (<https://www.ncbi.nlm.nih.gov/genome/annotation_euk/process/database>, accessed on 13 May 2022). To start the extraction of CDS, the gene name, email address, and a set of species must be selected. Optionally, the exclusion of intact start and stop codons and ORFs can be chosen as well. The CDS-extractor is accessible at <http://retrogenomics.uni-muenster.de/tools/CDS-extractor> (accessed on 13 May 2022). After starting the tool, a fasta results file with species names and accession numbers as headers is immediately downloadable. The aligner MUSCLE (<http://www.drive5.com/muscle/>, accessed on 13 May 2022) can be used for the subsequent multiple sequence alignment (version 5 [20]). After codon-wise gap removal, the alignment is ready for paPAML.

*2.2. paPAML*

The basic concept of our Perl command-line tool paPAML is to run different modules of codeml and HyPhy FEL in parallel processes, which minimizes the administrating time and maximizes the visibility of phylogenetic branches and codon positions that were exposed to natural selection. That is accomplished by simultaneously running independent codeml calculations of all automatically labelled individual tree topologies (foreground labelled by, e.g., one #1 mark per tree topology) for the branch- and branch-site-specific tests. This enables shorter total runtimes, especially on large multicore computer systems, without changing the basic algorithms of either codeml or HyPhy FEL. paPAML enables user-defined settings of the upper limit of parallel runs allowing for optimal adaptation to the computational environment. Furthermore, paPAML reduces preparation time for large datasets by automatically specifying ctl configurations for each test (default or entered command line parameters) and tree/tre-files and coordinates to run all subsets of tests. According to codeml requirements, the outgroup species must be placed as the first sequence in the alignment. Because PAML automatically removes positions with gaps in alignments and HyPhy treats such positions as ambiguous bases by default instead, prior manual removal of all such gap positions is advisable to apply codeml and HyPhy based on the same alignment. Correspondingly, the positions of detected positively or negatively selected sites relate directly to the modified alignment of the reference species (first sequence) and require a subsequent realigning to the original genes of individual species. Furthermore, stop codons need to be removed. The tree must be in Newick format. Figure 3 illustrates the input and output formats, an example command, and the parallelization concept.

**Figure 3.** Input format, basic command-line options, and output of paPAML. The standard input of codeml and HyPhy is a fasta sequence file of aligned CDS excluding the stop codon, a Newick tree/tre file representing the corresponding phylogenetic tree, and a ctl file prefilled with the seqfile name (e.g., “seqfile = ALIGNMENT.fasta and treefile = NEWICK.tre”). The command shown here *paPAML.pl -p 10* enables the usage of ten threads in parallel. The option [-d] allows the storage of directories containing the data from the subprocesses. The original input and output data, as well as the created ctl file can be accessed here. Test 1 represents the site models M1a/M2a and M7/M8. Test 2 executes the branch-site models and compares results for ω = 1 (fixed) versus ω > 1 (unfixed). Test 3 denotes the corresponding branch model. Test 4 evaluates site-specific incidences of negative and positive selection by HyPhy. The output file structure is shown at the bottom of the Figure.

paPAML allows a restart and continuation of aborted runs. Finally, paPAML screens the result files of each test to combine and summarize them in a final output file. This includes the automated calculation of p-values for the model comparisons via a chi-squared test using the extracted lnL values and degrees of freedom from both models. Furthermore, paPAML creates a tabular sequence overview. Each codon is connected to its extracted, site-specific result, making assigning selection pressures to sites in the original sequence easier. The summarized and structured output allows even inexperienced users to comb through copious amounts of data. The complete list of options for the command line is as follows:

```
VERSION 1.19
paPAML.pl -p threads [-f controlfiles] [-t tests] [-s significance] [-d] {codemlparams}
paPAML.pl -i [provides status information of runs in a new terminal window]
paPAML.pl -c [removes all temporal folders]
CTRL-c        [interrupts the run and all subprocesses]


WHERE
    runs         		- The number of parallel runs.
    controlfiles 	- A list of control files.  It is assumed they are named
                   	with a suffix "suffix".ctl!  If not given, all files with
                   	that suffix are used for calculation.
    tests        		- The used tests (1, 2, 3 or h for HyPhy) to run the data.
			    	They can be written like "1" or "12" (order is not important).
                   	(Default: 123h).
    significance 	- The maximum p-value to print marked trees.  Also used for
                   	printing BEB and HyPhy results.
                   	(Default: 0.05).
    -d           		- The original result directories (PAML, HyPhy) are stored.
    -i           		- Info about current runs.
    -c           		- Cleans all temporary folders.
    codemlparams 	- Additional parameters for the ctl file, if not provided
                   	the default parameters will be used (see below).
                   	(Example: -Mgene 9 -rho 34)

DEFAULT CODEML-PARAMETERS
    -CodonFreq     	2
    -Malpha        	0
    -Mgene         	0
    -RateAncestor  	0
    -Small_Diff    	0.5e-6
    -aaDist        	0
    -alpha         	0
    -cleandata     	1
    -clock         	0
    -fix_alpha     	1
    -fix_blength   	-1
    -fix_rho       	1
    -getSE         	0
    -icode         	0
    -method        	0
    -ndata         	1
    -omega         	1
    -outfile       	mlc
    -rho           	0
    -runmode       	0
    -seqtype       	  1
```

*2.3. Data Requirements (paPAML)*

Both codeml and HyPhy require aligned sequence files in the fasta format for each locus to be analyzed. These sequences need to be structured in codons with a base pair count divisible by three (for intact ORFs) and no stop codons. Because codeml and HyPhy deal differently with sequence gaps (codeml deletes all gap regions and HyPhy fills them with unspecific bases), it is also necessary to consistently remove all gap regions codon-wise. Additionally, both programs need a phylogenetic tree/tre file in the Newick format with all species represented in the alignment. Species names in this phylogeny must be equivalent to the sequence names in the fasta file. A codeml ctl config file is needed as a template for paPAML. The ctl file specifies the seqfile and treefile names (e.g., "seqfile = ALIGNMENT.fast" and "Treefile = NEWICK.tre"). All other PAML-specific parameters can either be specified by the user via the command line, or default settings are used (see command-line call: paPAML.pl). One must start paPAML in the directory containing the input files and ctl files.

*2.4. Necessary Resources (paPAML)*

For an optimal performance of paPAML, we advise using a multicore computer with a UNIX-based operating system such as Linux, FreeBSD, or macOS with multiple processors. The speed of analyses is multiplied depending on the number of threads. paPAML was developed and tested with Perl version 5.32 but theoretically works with other versions. It requires the following Perl modules: Proc::ProcessTable (version 0.59) and Statistics::Distribution (version 1.02). It also requires the preinstalled programs PAML (version 4.9j, <http://abacus.gene.ucl.ac.uk/software/paml.html>, accessed on 13 May 2022 [6]), HyPhy (version 2.5.36, <https://github.com/veg/hyphy/releases>, accessed on 13 May 2022 [11]) and their respective prerequisites. paPAML source code is available at github.com. The program accepts any length of sequences and number of species. However, processing time increases the larger the input data set becomes.

**3. Results and Discussion**

paPAML is especially useful for frequent applicants and inexperienced users of tests to determine selection pressures on sequences. It offers a simplified entry of data, compiles the essential tests for selection pressure, provides structured, compiled output files for the applied site models M1a/M2a and M7/M8, branch models, branch-site models, and HyPHy FEL with a list of all analyzed codons and labelled significance values for positive and negative selection. In addition to the result table, paPAML generates a fasta file with highlighted selected codons (uppercase letters) for each applied test to facilitate a subsequent MUSCLE [20] alignment against the original genes from any species of interest. Essentially parallelized running of the different models and their extensive sub-processes (individually labelled branches for the branch-site and branch models) results in substantial time saving, particularly for long sequences and numerous taxa.

This we demonstrated on a variety of example datasets. All the example datasets were assembled using the CDS-extractor, and were analyzed both via paPAML and in individual PAML codeml and HyPhy runs to test paPAML for the correct identification of sites and branches under selection pressures and to monitor differences in run time. Marked differences in computational runtimes and manageability of the data, especially for longer and more species-rich comparisons, are shown in Table 1.

**Table 1**: Runtime analysis results for example datasets.

| Dataset | Number of  Sequences | Number of  Codons | Overall  runtime paPAML  (min) | Longest runtime  codeml  (min) | Overall  runtime  codeml  (min) |
| --- | --- | --- | --- | --- | --- |
| GINS3 exonization | 3 | 39 | < 1 | < 1 | < 1 |
| CEBPE | 30 | 281 | 198 | 1205 | 3032 |
| Lysin | 25 | 120 | 115 | 405 | 867 |
| APOL | 20 | 312 | 79 | 447 | 1143 |

All test runs were performed with the default paPAML settings with 10 parallel runs (paPAML.pl -p 10). Because we embedded the original PAML and HyPhy modules into paPAML, we could confirm that compared to codeml (PAML version 4.9j) and HyPhy (HyPhy FEL version 2.5.26), all test runs led to identical selection pressure results. In general, the total selection analysis via paPAML, including all model comparisons, was about 3 to 6 times faster than the longest running codeml model and about 7 to 15 times faster than the entire processing time of these codeml runs.

*3.1. Four Examples of Test Data*

(1) A potentially exonized *Alu* element sequence in the GINS Complex Subunit 3 gene (GINS3). The process of exonization involves the recruitment of novel exons from, e.g., intronic mobile elements or transposed elements (TE) without an original protein-coding function (see Schrader and Schmitz [14,15]). Here, we tested for selective constraints on the novel TE exon cassette. Such novel exons usually follow a path from neutral to adaptive and purifying selection. The dataset contained orthologous sequences from three primate species (*Homo sapiens*, the olive baboon *Papio anubis*, and the Angola colobus *Colobus angolensis*) each with a length of 117 base pairs. Analysis of the GINS3 exonized region dataset finished in about 26 s. The overall runtime for all consecutive codeml model comparisons was 39 s, and the longest run of a single test took 18 s. Due to the low number of sequences and species involved, a runtime comparison for GINS3 was not very informative, but paPAML was still faster overall than the codeml analysis overall. The parallelization our program provides did not have a large effect on runtimes for small datasets in general but still is advantageous for such datasets by simplifying in- and output of the analyses.

Multiple sites in the potential GINS3 *Alu* exonization [15] appear to be under significant positive selection (Table 2). According to the site model, both the M1a/M2a and M7/M8 comparisons showed that the models allowing for positive selection fit the data better than their respective null models (p-value M1a/M2a = 0.035, p-value M7/M8 = 0.026). Also, eight sites were found to be significantly likely to be under positive selection in both comparisons (p-value < 0.05; codons 2, 8, 24, 25, 26, 28, 29, 37; Supplementary Table S1) in both comparisons. The branch-site model indicated that, except for codons 8 and 24, all of these sites have been under positive selection both in the branch leading to *Homo sapiens* and the branch leading to the other two species (see Figure 4 for significant selection on branches derived from the branch-site model). There were no significant results in the branch model and HyPhy FEL test.

**Figure 4:** Phylogenetic tree of the GINS3 dataset. Branches significantly likely to be under positive selection according to the branch-site models are marked orange (corresponding to Figure 2) and labelled with the p-values inferred by their respective model comparisons.

(2) The neutrophil associated CCAAT Enhancer Binding Protein Epsilon (CEBPE) transcription factor with 30 *Placentalia* sequences, each 843 base pairs long. The CEBPE gene significantly influences the development and function of neutrophils, the most abundant leukocyte subset in human blood, and is expected to be under selective forces [16] paPAML needed about 3.3 hours to finish the more extensive CEBPE dataset. By comparison, codeml’s most time-consuming model comparison took about 20 hours to complete, while the overall codeml analysis, if executed consecutively, would have taken over 50 hours.

Neither the site model nor the branch-site model detected sites under positive selection in the CEBPE gene. Instead, 68 of the 281 codons were subject to purifying selection according to the HyPhy FEL algorithm, which did not detect any positively selected sites. The branch model, on the other hand, found about 22 out of 50 branches with signals of positive selection, including the branch leading to *Homo sapiens*. This may indicate that while the gene is primarily subject to strong purifying selection and is most likely highly conserved, it was also regularly the focus of episodic positive selection during its phylogenetic history (Figure 5). Note that sites under positive selection could be identifiable by expanding the analyzed species spectrum to other representatives of mammals or vertebrates.

**Figure 5:** Phylogenetic tree of the CEBPE dataset. Branches significantly likely to be under positive selection according to the branch models are marked green (corresponding to Figure 2) and labelled with the p-values inferred by their respective model comparisons.

(3) The classical example for positive adaptive selection – the abalone sperm lysin gene. The dataset consisted of orthologous lysin sequences with a length of 360 base pairs from 25 species of the sea snail genus Haliotidae (abalones) [21]. The paPAML analysis of the lysin dataset finished in approximately 115 mins. By comparison, the longest running codeml test (the branch-site model comparison) took almost 405 min to complete. If the models were used consecutively, the overall run of all the model comparisons in codeml would have taken about 14.5 hours. Ubiquitous signals of positive selection on multiple branches as well as negative selection on several sites were found in the lysin example dataset. All significantly supported positive selection pressures on branches are shown in Figure 6. The site model showed a remarkably high fit of the positive selection model to the dataset (p < 0.000001 for both M1a/M2a and M7/M8) as well as 17 of 120 sites under significant positive selection, although codon 114 was only detected with the M7/M8 model comparison. The HyPhy FEL algorithm found an overlap with positively selected sites and that codons 24, 32, 75, 114, and 120 (Supplementary Table S1) were positively selected. On the other hand, it also found codons 33, 63, 94, 111, 115, and 119 (Supplementary Table S1) to be under positive selection, which the site model did not confirm. Such differences between the models are to be expected due to their different methods of calculating selection pressures. The branch model detected 11 out of 47 branches to be under significant positive selection with ω values averaged over sites. By comparison, the branch-site model detected specific sites under selection in 9 branches. They were not, however, entirely overlapping, with the branch leading to *Haliotis varia* and the one to *Haliotis siboldii* found to be positively selected only by the branch model. Additionally, the branch-site model detected positively selected sites in the branch leading to *Haliotis kamtschatkana* and three others that were not found to be under significant positive selection pressure by the branch model. This was also expected, as the branch model searches for selection signals by averaging ω-values over sites, while the branch-site model allows sites to have different ω -values. About 26 codons were also found to be under positive selection pressure only in specific branches (for example, codon 15 in the branch leading to *Haliotis kamtschatkana*). Lastly, the HyPhy FEL algorithm revealed 15 codons under purifying selection (Supplementary Table S1). Interestingly, one of them, codon 45, seems to be under positive selection in the branch leading to *Haliotis cyclobates*. Because this codon is otherwise highly conserved, averaged over the entire phylogeny this might indicate a potentially adaptive change unique to the species, which might have been missed without the added contrast of the detection of negatively selected sites.

**Figure 6:** Phylogenetic tree of the lysin dataset. Branches significantly likely to be under positive selection according to the branch-site and branch models are marked orange and green, respectively (corresponding to Figure 2) and labelled with the p-values inferred by their respective model comparisons. This includes branches with significant LRT results in the branch-site model but without sites under positive selection.

(4) The apolipoprotein encoding genes APOL1-4, which demonstrably contains many codon positions under positive selection pressure [18]. The paPAML analysis of the APOL dataset, which contains fewer but longer sequences than our other examples, was completed in 79 minutes. The APOL codeml analysis took about 19 hours overall, and the most time-intensive model comparison about 7.5 hours. The APOL1-4 gene data showed signs of both positive and negative selection at many sites (Figure 7). Both site model comparisons indicated a highly significant fit to the data (p < 0.00001 for both M1a/M2a and M7/M8). 22 of the 312 codons of the analyzed sequences were identified to be under positive selection by both models, while codons 8, 193, and 263 were found to be under adaptive selection only by the M7/M8 comparison (Supplementary Table S1). HyPhy FEL also showed a large overlap with the site models in identifying positively selected sites; of the 12 codons found, numbers 165, 209, 247, and 266 were not detected by the codeml models (Supplementary Table S1). According to the branch-site model, codons 88 and 163 underwent episodic positive selection in the branch leading to the APOL4 of *Chlorocebus sabaeus* (green monkey), while the same applies for the codon 36 isoform 4 branch of *Gorilla gorilla* (gorilla) (Supplementary Table S1). The branch model was able to find signals of positive selection in 5 different branches, including the APOL4 *Chlorocebus sabaeus* branch, but not the APOL3 *Gorilla gorilla* branch. Similar to the results of the abalone sperm lysin dataset, this difference between the two models is likely caused by the calculating differences inherent in the branch and the branch-site models. Lastly, HyPhy FEL found 10 sites under purifying selection without any overlap with codons detected by the other model comparisons. It is of note that HyPhy FEL is a maximum likelihood-based test like the site model comparisons, which means that we do not expect it to find purifying selection acting on codons that the site models predict to be positively selected.

**Figure 7:** Phylogenetic tree of the APOL1-4 dataset. Branches significantly likely to be under positive selection according to the branch-site models (orange) and branch models (green) (corresponding to Figure 2) are labelled with the p-values inferred by their respective model comparisons. This includes branches with significant LRT results in the branch-site model but without sites under positive selection.

Such selection analyses are directly open for subsequent functional studies, for example, applied in Müller et al. [18]. From the previously detected 27 positive selected sites using PAML [18], we could reconstruct most of them with paPAML (21) plus seven previously unrecognized cases (Supplementary Data S2). The difference is probably due to the usage of updated alignments and maximum likelihood-caused variation. All used sequences, tree- and control files are available as Supplementary Data.

**Table 2:** Results of the selection analyses. The number of branches under positive selection detected by the branch-site model did not include branches with significant LRT results but without sites under positive selection. Overlaps between found sites were counted for all models with which they were found.

| Data set | Number of codons in sequence | Number of positively selected codons  (Site models) | Number of positively selected codons (branch-site model) | Number of positively selected codons (HyPHY FEL) | Number of negatively selected codons  (HyPhy FEL) | Number of branches under positive selection |
| --- | --- | --- | --- | --- | --- | --- |
| GINS3 exonization | 39 | 8 | 0 | 0 | 0 | 2 (branch-site model) |
| CEBPE | 281 | 0 | 0 | 0 | 68 | 22 (branch model) |
| Lysin | 120 | 17 | 32 | 11 | 15 | 11 (branch model),  9 (branch-site model) |
| APOL1-4 | 312 | 25 | 3 | 12 | 12 | 5 (branch model)  2 (branch-site model) |

*3.2. Comparison to Other Selection Analysis Tools*

The results of original PAML analyses are difficult to parse for newcomers, not parallelized and therefore very time-inefficient, as well as not easy to include in high-throughput data analyses. It is necessary to manually calculate LRT results and edit ctl configuration files to change parameters and models. Our program is designed to reduce the time of a complete selection analysis as much as possible without changing the underlying assumptions of the base models of either PAML or HyPhy FEL. This is exemplified by our comparative paPAML and codeml runs, which produced identical results for all four of our example datasets, while paPAML reduced the processing time considerably. It also simplifies the results and allows both a form of high-throughput analysis and the continuation of aborted runs if needed. Other programs have been created to alleviate these problems as well, including but not limited to EasyCodeML [22], LMAP [23], and BlastPhyMe [24], all of which also automatize PAML LRT calculations, allow for limited multithreading, and summarize the results. BlastPhyME and EasyCodeML even provide a graphical user interface (GUI). EasyCodeML also offers drag-and-drop functionality and visual labelling of tree branches via single clicks for easy use. That, however, severely limits the ability of these programs to do high-throughput analysis, as each run requires the use of the GUI, and no command-line version for integration into larger pipelines is available. All four programs also provide different multithreading methods: LMAP and BlastPhyMe allow multiple datasets to be run simultaneously by mapping codeml tasks to different cores. In contrast, EasyCodeML's multithreading occurs in the site model's analysis. paPAML does this by splitting the analysis of each tree topology in the branch and branch-site models, and then running as many codeml tasks as possible on the number of cores previously specified by the user. This also includes parallelizing multiple datasets by starting the first task of a new dataset once a core is no longer working on a task of the previous one. The parallelization does present limits, specifically that a site-specific test run is precisely as fast as in base codeml. It is not trivial to compare these different parallelization methods with one another, though each of them requires large multicore systems to enable significant reductions in processing time. Because we chose to focus here on these specific forms of selection analysis, further tests and models of PAML were not automatically included in paPAML. This includes the clade model, integrated into the other three programs. But, in contrast to, for example, BlastPhyMe, it is possible to change codeml parameters through paPAML’s command line, so alternate tests can still be executed using the program. Still, they might not be supported by our output summary. None of the other three programs or PAML itself include a test for negatively selected sites, limiting them to analyses searching only for positive selection. No other program includes a tabular overview of the site-specific results mapped to the codons of a sequence. This allows easy parsing of the results of all site-specific models and should be of specific help for inexperienced users. Lastly, any restrictions of either the codeml or HyPhy FEL tests apply to paPAML and the three other programs. The paPAML results compile the original outcome of all individual tests. It is the task of the user to interpret such results and compare them. Users should be aware that the applied selection analyses are based on maximum likelihood estimations, which can cause slight variations in the results even if the same parameters are used.

**4. Conclusions**

Here we provide a complete solution from extracting orthologous CDS to calculating and visualizing significance values of positively and negatively selected codons and phylogenetic branches applying the CDS-extractor and paPAML. Different site, branch-site, and branch models provide the most common sources to detect positively selected sites and branches in phylogenetic trees. With paPAML, this is now expanded by a HyPhy test for negatively selected sites as well. An automated parallelization of different tests and analysis of all possible foreground tree topologies within tests, provides a huge timesaver. The necessary comparisons of ω ratios and the calculation of significance values are compiled in a structured outfile. The CDS-extractor is easily applicable by using a public server at <http://retrogenomics.uni-muenster.de/tools/CDS-extractor> (accessed on 13 May 2022). paPAML can be downloaded from <https://github.com/RetroWWU/paPAML> (accessed on 13 May 2022) and installed as a stand-alone program on any UNIX computer, preferably with multiple cores and large memory capacity and with the necessary environment, including the PAML and HyPhy programs.

**Supplementary Materials:** All data are present in Supplementary Data, Supplementary Table S1 or deposited at <https://github.com/RetroWWU/paPAML> (accessed on 13 May 2022).

**Author Contributions:** Conceptualization R.S. and J.S., software design paPAML N.G., software design CDS-extractor A.P. and N.G., data collection and analyses R.S. and L.O., supervision J.S. and O.S., primary writing R.S. and J.S. L.O. and O.S. contributed discussion, editing, and writing. Graphics from L.O. and J.S. All authors have read and agreed to the published version of the manuscript.

**Funding:** This work was supported by the Deutsche Forschungsgemeinschaft (DFG, German Research Foundation) - RTG2220 - project number 281125614 to JS.

**Acknowledgement:** We thank Delf-Magnus Kummerfeld and Liliya Doronina for their comments on the manuscript. For editorial assistance, we thank Marsha Bundman. We acknowledge support from the Open Access Publication fund of the University of Münster.

**References**

1. Lander, E.S.; Linton, L.M.; Birren, B.; Nusbaum, C.; Zody, M.C.; Baldwin, J.; Devon, K.; Dewar, K.; Doyle, M.; FitzHugh, W.; et al. International human genome sequencing consortium. Initial sequencing and analysis of the human genome. *Nature* **2001**, *409*, 860-921.

2. Castro-Chavez, F. The rules of variation: Amino acid exchange according to the rotating circular genetic code. *J. Theor. Biol.* **2010**, *264*, 711-721.

3. Strachan, T.; Read, A.P. Human molecular genetics. 2nt ed. Wiley, New York, 1999, p. 576.

4. Nielsen, R.; Yang, Z. Likelihood models for detecting positively selected amino acid sites and applications to the HIV-1 envelope gene. *Genetics* **1998**, *148*, 929-936.

5. Yang, Z.; Nielsen, R.; Goldman, N.; Pedersen, A.M. Codon-substitution models for heterogeneous selection pressure at amino acid sites. *Genetics* **2000**, *155*, 431-449.

6. Yang, Z. PAML 4: Phylogenetic analysis by maximum likelihood. Mol. Biol. Evol. **2007**, *24*, 1586–1591.

7. Yang, Z.; Wong, W.S.W.; Nielsen, R. Bayes empirical Bayes inference of amino acid sites under positive selection, Mol. Biol. Evol. **2005**, *22*, 1107–1118.

8. Zhang, J.; Nielsen, R.; Yang, Z. Evaluation of an improved branch-site likelihood method for detecting positive selection at the molecular level. *Mol. Biol. Evol.* **2005**, *22*, 2472-2479.

9. Yang Z. Likelihood ratio tests for detecting positive selection and application to primate lysozyme evolution. *Mol. Biol. Evol.* **1998**, *15*, 568-573.

10. Yang, Z.; Nielsen, R. Synonymous and nonsynonymous rate variation in nuclear genes of mammals. *J. Mol. Evol.* **1998**, *46*, 409-418.

11. Pond, S.L.K.; Frost, S.D.W.; Muse, S.V. HyPhy: hypothesis testing using phylogenies. *Bioinformatics* **2005**, *21*, 676–679.

12. Pond, S.L.K.; Frost, S.D.W. Not so different after all: A comparison of methods for detecting amino acid sites under selection. Mol. Biol. Evol. **2005**, *22*, 1208–1222.

13. Hartig, G.; Churakov, G.; Warren, W.; Brosius, J.; Makałowski, W.; Schmitz, J. Retrophylogenomics place tarsiers on the evolutionary branch of anthropoids. *Sci. Rep.* **2013**, *3*, 1756.

14. Schrader, L.; Schmitz, J. The impact of transposable elements in adaptive evolution. *Mol. Ecol.* **2019**, *28*, 1537-1549.

15. Zhang, F.; Raabe C.A.; Cardoso-Moreira M.; Brosius, J.; Kaessmann H.; Schmitz, J. ExoPLOT: Advance in differential expression of alternative human exons. *Submitted*.

16. Pinheiro, D.; Mawhin, M.A.; Prendecki, M.; Woollard, K.J. In-silico analysis of myeloid cells across the animal kingdom reveals neutrophil evolution by colony-stimulating factors. *Elife* **2020**, *9*, 60214.

17. Massingham, T.; Goldman, N. Detecting amino acid sites under positive selection and purifying selection. *Genetics* **2005**, *169*, 1753–1762.

18. Müller, D.; Schmitz, J.; Fischer, K.; Granado, D.; Groh, A.C.; Krausel, V.; Lüttgenau, S.M.; Amelung, T.M.; Pavenstädt, H.; Weide, T. Evolution of renal-disease factor APOL1 results in cis and trans orientations at the endoplasmic reticulum that both show cytotoxic effects. *Mol. Biol. Evol.* **2021**, *38*, 4962-4976.

19. Cock, P.J.A.; Antao, T.; Chang, J.T.; Chapman, B.A.; Cox, C.J.; Dalke, A.; Friedberg, I.; Hamelryck, T.; Kauff, F.; Wilczynski, B.; de Hoon, M.J.L. Biopython: freely available python tools for computational molecular biology and bioinformatics. *Bioinformatics* **2009**, *25*, 1422–1423.

20. Edgar R.C. MUSCLE v5 enables improved estimates of phylogenetic tree confidence by ensemble bootstrapping. *bioRxiv* **2021**, https://doi: 10.1101/2021.06.20.449169.

21. Lee, Y.H.; Ota, T.; Vacquier, V.D. Positive selection is a general phenomenon in the evolution of abalone sperm lysin. *Mol. Biol. Evol.* **1995**, *12*, 231-238.

22. Gao, F.; Chen, C.; Arab, D.A.; Du, Z.; He, Y.; Ho, S.Y.W. EasyCodeML: A visual tool for analysis of selection using CodeML. *Ecol. Evol.* **2019**, *9*, 3891-3898.

23. Maldonado, E.; Almeida, D.; Escalona, T.; Khan, I.; Vasconcelos, V.; Antunes, A. LMAP: Lightweight multigene analyses in PAML. *BMC Bioinformatics* **2016**, *17*, 354.

24. Schott, R.K.; Gow, D.; Chang, B.S.W. BlastPhyMe: A toolkit for rapid generation and analysis of protein-coding sequence datasets. *BioRxiv* **2016**, https://doi.org/10.1101/059881.
